# Lichen nitidus

**DOI:** 10.11604/pamj.2019.32.39.13564

**Published:** 2019-01-22

**Authors:** Fatima-Zahra Agharbi

**Affiliations:** 1 Tetouan Regional Hospital Center, Tetouan, Morocco

**Keywords:** Lichen, nitidus, papules, lichenoide, dermatosis

## Image in medicine

Lichen nitidus (LN) is chronic papulosquamous disorder characterized by multiple, 1-2 mm, flesh-colored, shiny, dome-shaped papules. Its incidence is 0.034% in a study of skin diseases in blacks over a 25-years period. Skin lesions classically involves the genitalia, upper extremities, chest and abdomen. Infrequently, the lower extremities, palms, soles, face, nails, and mucous membranes may be affected. Majority of cases are common in children and young adults. Various clinical variants of lichen nitidus are - linear, confluent, vesicular, haemorrhagic, spinous follicular, perforating, generalised, palmar and plantar. We report the observation of a 6-month-old infant who presented translucent papules of the back of the hands whose histological study was in favor of a lichen nitidus.

**Figure 1:**
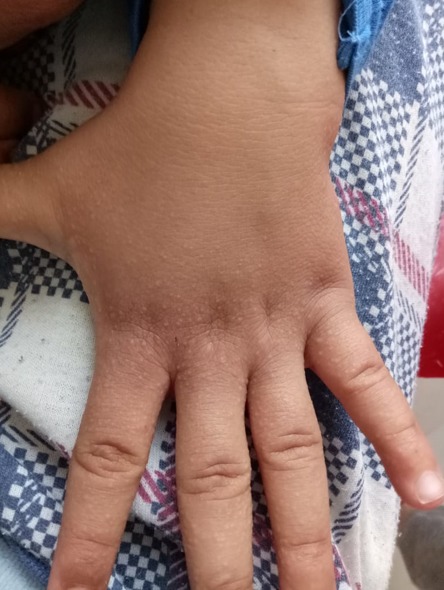
Translucent papules of the back of the hands

